# Visualized knowledge mapping on the current research trends and emerging areas of neuroimmune modulation in the past two decades

**DOI:** 10.3389/fimmu.2025.1671578

**Published:** 2025-12-05

**Authors:** Xu Han, Yan Zhou, Xiyao Gu

**Affiliations:** 1 Department of Radiology, Renji Hospital, Shanghai Jiao Tong University School of Medicine, Shanghai, China; 2 Department of Anesthesiology, Renji Hospital, Shanghai Jiao Tong University School of Medicine, Shanghai, China; 3 Key Laboratory of Anesthesiology (Shanghai Jiao Tong University), Ministry of Education, Shanghai, China

**Keywords:** neuroimmune modulation, crosstalk, web of science, pubmed, visualized knowledge mapping, citespace, VOSviewer, co-citation analysis

## Abstract

**Aim:**

Reference data analysis and visualization methods were applied to identify current knowledge mapping methods in the neuroimmune modulation research field.

**Methods:**

A comprehensive search of publications within the field of neuroimmune modulation in the Web of Science Core Collection database and PubMed from 2005–2024 was conducted. The reference type was restricted to articles and reviews in the WOSCC database and clinical trials in PubMed. The data were visualized and analyzed via visualization and bibliometric tools, including CiteSpace and VOSviewer.

**Results:**

A total of 5,280 publications were included in the field of neuroimmune modulation. The United States ranked first as the most influential country in terms of both publication number and academic influence. Harvard University had the greatest impact on institutions. The leading scientists in the neuroimmune modulation field have focused on the mechanism at both the peripheral and central levels. Citation analysis and cocitation analysis revealed that acute kidney injury, neuropathic pain, and neuronal regulation are key focal points in neuroimmune research. Furthermore, keyword analysis revealed that “inflammation”, “neuroinflammation”, “microglia”, “cytokines”, “cholinergic anti-inflammatory pathway” and “neuroimmune” had more than 200 co-occurrences and represented research hotspots in this field. A total of 73 clinical trials were identified that targeted neuroimmune modulation to treat diseases. These studies highlighted the central role of the neuroimmune network in diseases, with a particular emphasis on innovative therapies that regulate inflammation through the cholinergic system. Further exploration is needed to develop precise intervention strategies targeting specific cytokines.

**Conclusions:**

This analysis provides comprehensive knowledge mapping in the current research field of neuroimmune modulation. The trends in the field include mechanism studies combined with neuronal regulation. Neuroimmune crosstalk might provide new therapeutic targets for treating nervous system diseases.

## 1 Introduction

The interaction between the nervous and immune systems, referred to as neuroimmune modulation, is a bidirectional process facilitated by neurotransmitters, hormones, cytokines, and neural innervation (1, 2). Nerve-released neurotransmitters and neuromodulators interact with immune cell receptors, whereas endocrine-secreted hormones modulate immune responses. Cytokines influence the nervous system, and neural innervation of lymphoid organs directly affects immune cells, creating a reciprocal relationship between these systems.

This neuroimmune interaction is vital for sustaining both healthy and diseased states. During immune defense, it harmonizes responses to pathogens while curbing excessive inflammation (3). In autoimmune diseases, disruption of this neuroimmune interplay accelerates disease progression (4). Under neurodegenerative conditions, neuroinflammation intensifies neuronal injury (5). Notably, in mental health disorders, a functional link between the nervous and immune systems emerges, with chronic low-grade inflammation implicated in conditions such as depression. Consequently, unraveling the dynamics and mechanisms of this interaction is pivotal for developing novel therapies for diverse diseases.

In recent years, studies on neuroimmune regulation have proliferated significantly. Despite the vast and expanding literature in this domain, scientific approaches via bibliometric analyses capable of fully mapping research trends and elucidating knowledge structures remain scarce. While existing systematic reviews and meta-analyses offer valuable insights, they often focus on narrow aspects, limiting their capacity to provide a holistic perspective. Research indicates that comprehensive analyses of knowledge architecture and emerging research focal points are particularly advantageous for newcomers and early-stage investigators (6). Against this backdrop, bibliometric methodologies have gained traction as dual-purpose tools—facilitating both quantitative assessments of scientific output and qualitative evaluations of developmental trajectories. Moreover, advancements in information technology have propelled the adoption of visualization software such as CiteSpace and VOSviewer, which are now routinely employed across disciplines such as neuroscience, oncology, and pediatrics (7–9).

This study seeks to conduct a comprehensive bibliometric analysis of the scholarly literature on neuroimmune modulation spanning the past two decades (2005–2024). The analysis aims to map key contributors, assess the current research landscape, and outline potential avenues for future inquiry into the neuroimmune modulation research field.

## 2 Materials and methods

### 2.1 Data source and search strategy

**Figure 1:**
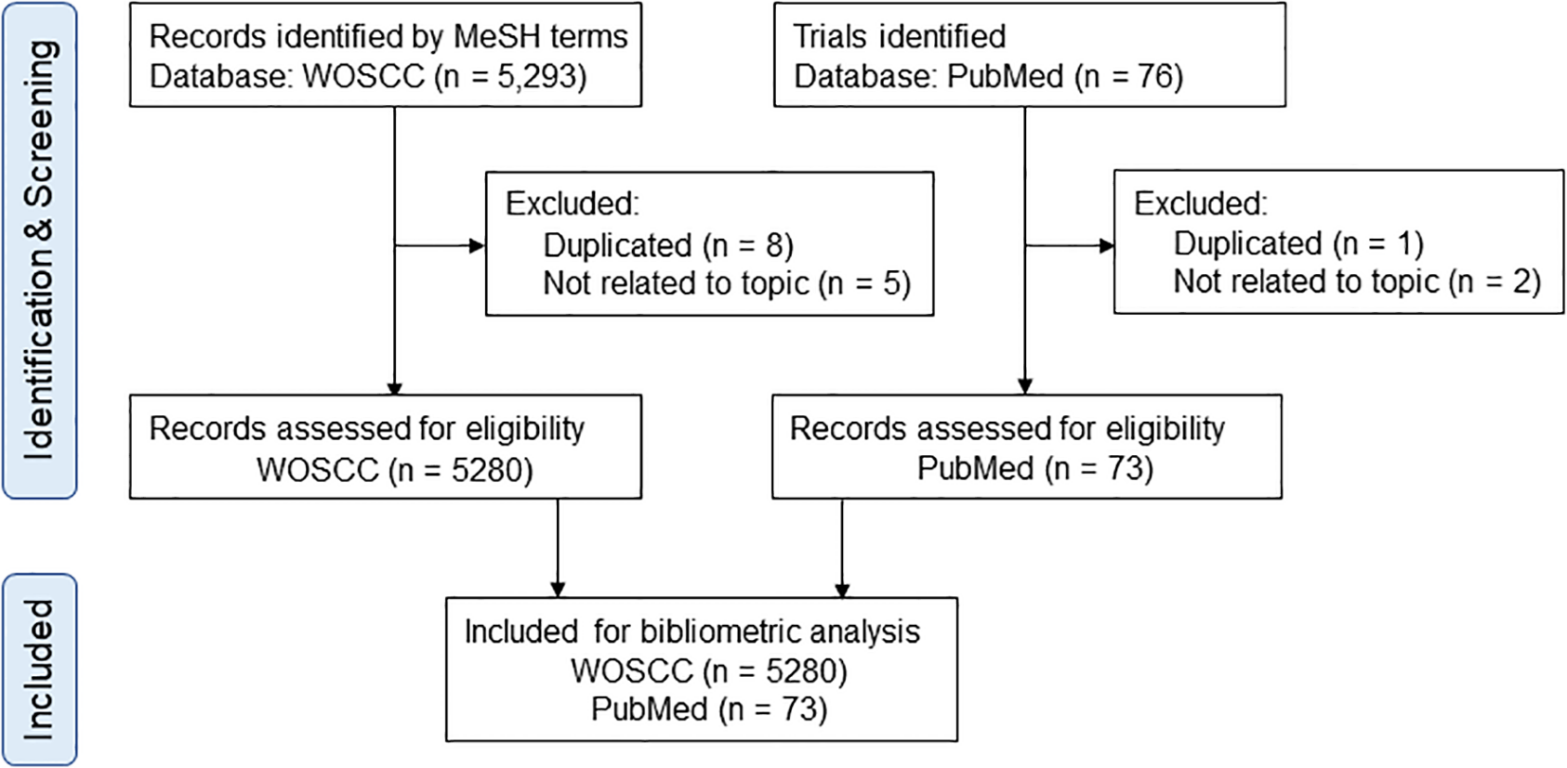
The flow diagram of references identification and screening.

We conducted a comprehensive search of the Web of Science Core Collection (WoSCC) database (10, 11), the Science Citation Index Expanded (SCI-E) edition and the PubMed (pubmed.ncbi.nlm.nih.gov) database. The retrieval strategy used in our study was as follows. The initial search phrase “neuroimmune modulation” was selected to align with the journal’s thematic focus. To ensure comprehensive coverage of relevant literature, we expanded our search by incorporating Medical Subject Headings (MeSH) terms associated with this concept (MeSH Unique ID: D015213). This approach, supported by bibliometric analyses, enabled the inclusion of a broader range of references pertinent to the research theme (12). The topic was set as “neuroimmunomodulation” or “neuro-immunomodulation” or “neuro immunomodulation” or “neuro-immune Interaction” or “neuro-immune Communication” or “neuro immune Interaction” or “neuro immune Communication” or “neuro-immune Axis” or “neuro immune Axis” or “neuroimmune Mechanism” or “neuroimmune Process” or “neuroimmune Interaction” or “neuroimmune Communication” or “neuroimmune Axis” or “cholinergic Anti-inflammatory Pathway” or “cholinergic Anti-inflammatory Pathway” or “vagal Anti-inflammatory Pathway” or “vagal Anti-inflammatory Pathway” or “vagal-immune Interaction” or “vagal immune Interactions”. The retrieval time ranged from January 2005 to December 2024, the literature types were “article” and “review”, and the language was “English” only. For the PubMed database, the topics and retrieval times were the same, and the literature types were restricted to “clinical trial” and “randomized controlled trial”. To minimize bias arising from frequent database updates, all literature retrieval and data downloads were completed on a single day, April 22, 2025. The retrieved references were independently screened by XH and YZ to assess their relevance to the topic. Discrepancies in relevance assessments between the researchers were resolved by XYG. References unrelated to the study topic were subsequently discarded (**Figure 1**).

### 2.2 Data processing

The dataset, including complete publication records and corresponding citations, was extracted for detailed examination. The analytical tools used included Microsoft Excel (version 2019), CiteSpace (version 6.2. R4), and VOSviewer (version 1.6.20).

Microsoft Excel was leveraged for its robust capabilities in data visualization (13). In this study, the tool facilitated the creation of charts and graphs to illustrate trends and insights, such as annual publication volumes, the prolific output of the top 10 contributing countries/regions, and a global map depicting research contributions by country/region on the basis of publication counts.

CiteSpace, a prominent bibliometric tool, was employed to map the research field’s evolutionary trends, identify emerging hotspots, and anticipate future trajectories (14). Specifically, it facilitated the visualization of collaborative networks among countries/regions, institutions, and authors while enabling cocitation analysis of references and burst detection to highlight sudden shifts in scholarly focus.

VOSviewer is a critical bibliometric tool developed by Professor van Eck and Waltman (15). In this study, it was used to visualize keyword co-occurrence networks, reveal thematic clusters, and analyze temporal patterns in keyword usage, thereby tracing evolving research focus areas.

These bibliometric tools were employed to comprehensively visualize and analyze data on neuroimmune modulation, enabling the exploration of research trends, core themes, and emerging hotspots. The goal was to derive actionable insights into the field’s trajectory, collaborative dynamics, and future priorities.

## 3 Results

### 3.1 Publication outputs

**Figure 2:**
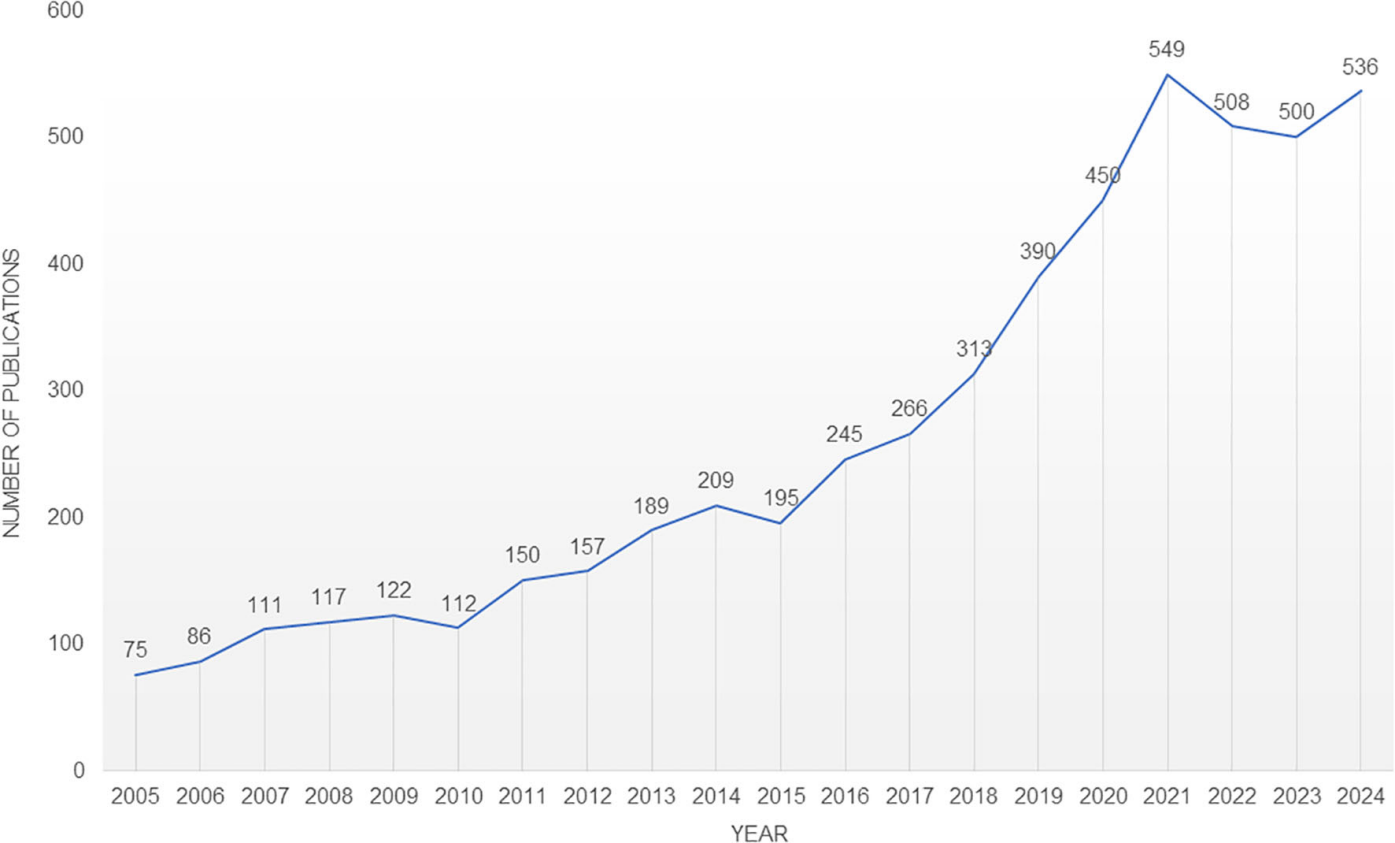
The annual number of publications in the neuroimmune modulation research field from 2004–2024.

The total identified number of publications (5,280) serves as a key metric for tracking trends in neuroimmune modulation research from the WoSCC database. Of these, 3,454 were original articles, and 1,826 were reviews, reflecting robust scholarly engagement. **Figure 2** illustrates the annual publication trajectory, which shows steady growth from 2005–2024, with a peak of 549 publications in 2021 (**Figure 2**). Despite minor fluctuations, this upward trend underscores the field’s expanding relevance and increasing interest over time.

### 3.2 Basic knowledge structures of the neuroimmune modulation field

#### 3.2.1 Analysis of the most productive countries/regions

**Figure 3:**
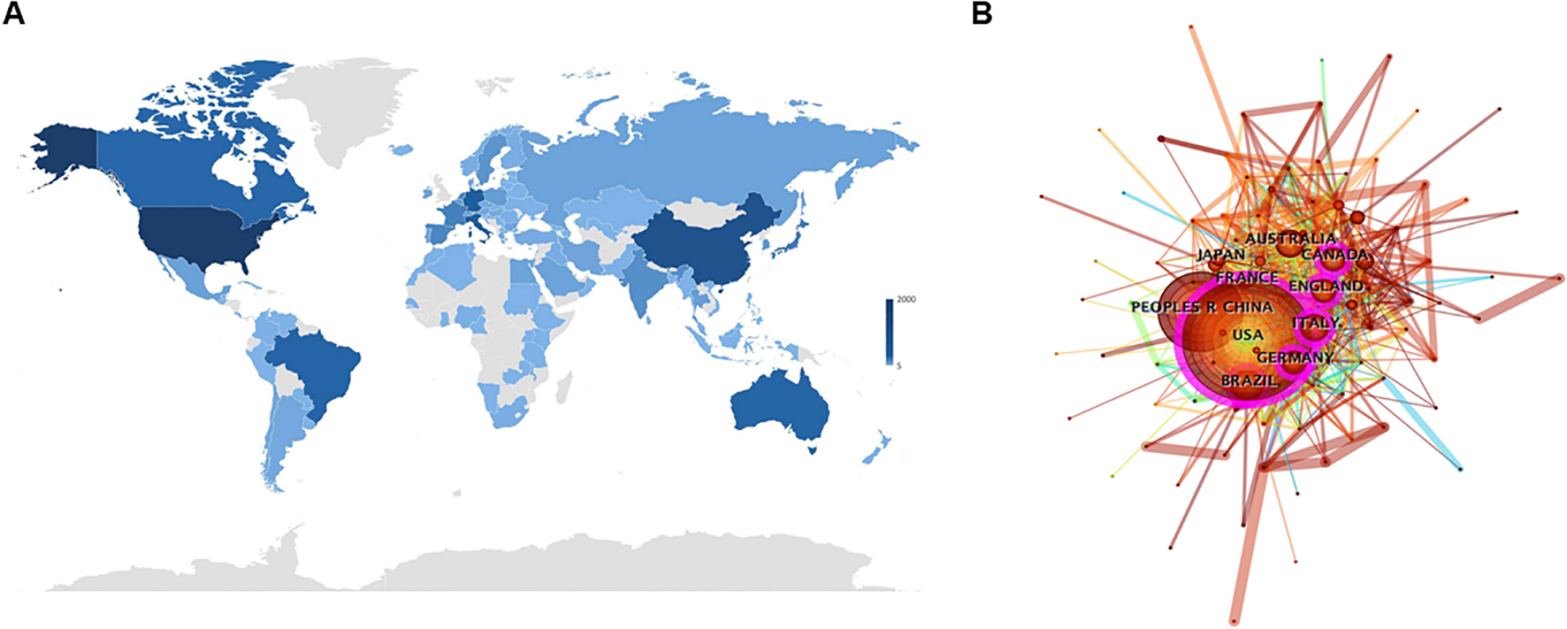
**(A)** Publication counts of each country/region on a world map. A deeper color indicates a greater number of publications. **(B)** Visualized mapping of the collaboration analysis among countries/regions.

**Figure 3A** depicts the global distribution of publications via a color-coded world map, with North America, Western Europe, and East Asia dominating contributions. The United States topped the list with 2,044 documents, followed by P. R. China (922), Germany (368), Italy (351), and Brazil (280) (**Table 1**). **Figure 3B** leverages CiteSpace to visualize collaborative networks among 106 countries/regions connected by 835 links (density = 0.15), highlighting active international research partnerships.

**Table 1:** Top 10 country/region, institution, and authors in terms of publication.

Rank	Country/region	Publications	Institution	Publications	Authors	Publications
1	USA	2044	Harvard University	185	Maes, Michael	62
2	China	922	University of California System	181	Tracey, Kevin J	34
3	Germany	368	Harvard University Medical Affiliates	152	Deak, Terrence	29
4	Italy	351	Harvard Medical School	143	Bakheet, Saleh A	24
5	Brazil	280	University of Texas System	125	Attia, Sabry M	24
6	Australia	278	Universidade de Sao Paulo	112	Nadeem, Ahmed	24
7	Canada	268	University System of Ohio	109	Ahmad, Sheikh F	24
8	England	249	Institut National de la Sante et de la Recherche Medicale (Inserm)	108	Ansari, Mushtaq A	22
9	Japan	199	University of London	93	Ulloa, Luis	22
10	France	179	US Department of Veterans Affairs	90	Chiu, Isaac M	22

Betweenness centrality (BC) was applied to evaluate the network importance of countries, with values >0.1 indicating central hubs (purple-ring markers) (16). The United States (BC = 0.33), England (0.2), Italy (0.16), Brazil (0.13), Germany (0.12), and Canada (0.12) surpassed this threshold, highlighting their pivotal roles in connecting collaborative networks. While regional partnerships, especially in North America and Western Europe, are evident, global cooperation remains underdeveloped, underscoring the need for expanded transnational research collaboration.

#### 3.2.2 Analysis of the most prolific institutions

**Figure 4:**
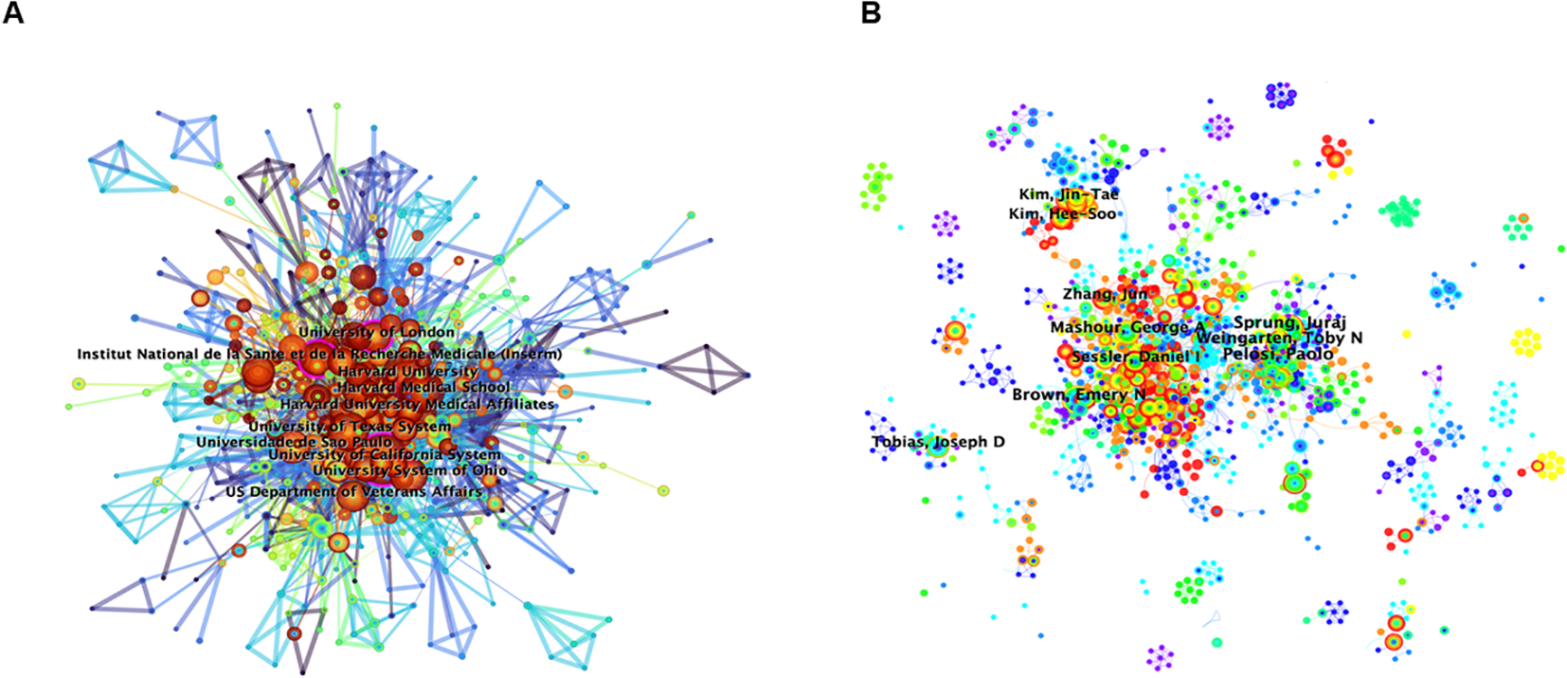
**(A)** Visualized mapping of the collaboration analysis among research institutions. **(B)** Visualized mapping of author collaboration analysis (via CiteSpace).

The CiteSpace-generated institutional network comprised 703 nodes, which indicated institutions, and 2,816 links, which indicated collaborations (**Figure 4A**). The top five contributors to neuroimmune modulation research were all U.S. based, including Harvard University (185 publications), the University of California System (181), Harvard Medical Affiliates (152), Harvard Medical School (143), and the University of Texas System (125). (**Table 1**). All five are based in the United States, highlighting its leading role in this field. Harvard University has the highest publication volume and a broad range of research topics related to neuroimmune modulation. Institutions with high intermediary centrality, such as the University of California System (0.17), the Institut National de la Sante et de la Recherche Medicale (Inserm) (0.15), the University of London (0.13) and the Karolinska Institutet (0.1), were crucial hubs for fostering international collaboration. Despite these hubs, transnational institutional cooperation remains minimal, underscoring the need for strengthened global partnerships.

#### 3.2.3 Analysis of influential authors

In neuroimmune modulation research, CiteSpace identified 7,062 authors and 22,177 collaborations, reflecting robust scholarly activity and frequent interdisciplinary partnerships (**Figure 4B**). Author productivity, as shown in **Table 1**, was dominated by Michael Maes (62 publications), a highly cited expert in neuropsychiatry with an H-index exceeding 120 and over 1,000 career publications. The work of Maes spans mental health biomarkers and neuroimmune disorders (17–21). Kevin J. Tracey ranked second, known for groundbreaking discoveries in inflammation, including HMGB1 mediators and bioelectronic medicine. As President of the Feinstein Institutes and Dean of the Elmezzi Graduate School, his research bridges immunology and neuroscience, with impacts on sepsis treatment and inflammatory reflex mechanisms (22–27). Terrence Deak, third in publication volume, focuses on neuroendocrine–immune interactions and early-life stress effects on brain function, using rodent models to dissect circuit-level changes linked to aging and social behavior. His recent work emphasized age-related social dysfunction mechanisms (28–31).

### 3.3 Overview of research hotspots and frontiers

#### 3.3.1 Analysis of highly cited studies

Citation analysis is fundamental to bibliometric studies, and despite some debate about its significance, citation counts are commonly viewed as indicators of a publication’s influence (32). Higher citation frequencies often reflect greater academic standing within a field (33). **Table 2** highlights the seminal works that have garnered substantial attention, with each of the top 10 most-cited papers amassing over 820 citations (34–43).

**Table 2:** Top 10 most cited publications.

Citations	Title	Source	First author	Publication year
1570	The Role of Short-Chain Fatty Acids From Gut Microbiota in Gut-Brain Communication	Frontiers in Endocrinology	Silva, Ygor Parladore (34)	2020
1568	Neuroglial activation and neuroinflammation in the brain of patients with autism	Annals of Neurology	Vargas, DL (35)	2005
1430	Neuropathic Pain: A Maladaptive Response of the Nervous System to Damage	Annual Review of Neuroscience	Costigan, Michael (36)	2009
1121	Transferring the blues: Depression-associated gut microbiota induces neurobehavioural changes in the rat	Journal of Psychiatric Research	Kelly, John R. (37)	2016
1101	Experimental autoimmune encephalomyelitis (EAE) as a model for multiple sclerosis (MS)	British Journal of Pharmacology	Constantinescu, Cris S. (38)	2011
940	Principles and clinical implications of the brain-gut-enteric microbiota axis	Nature Reviews Gastroenterology & Hepatology	Rhee, Sang H. (39)	2009
896	The Amyloid-β Pathway in Alzheimer's Disease	Molecular Psychiatry	Hampel, Harald (40)	2021
892	Neurotransmitter modulation by the gut microbiota	Brain Research	Strandwitz, Philip (41)	2018
859	Stimulation of the vagus nerve attenuates macrophage activation by activating the Jak2-STAT3 signaling pathway	Nature Immunology	de Jonge, WJ (42)	2005
824	Interleukin-1 and neuronal injury	Nature Reviews Immunology	Allan, SM (43)	2005

The top-ranked paper, “The Role of Short-Chain Fatty Acids from the Gut Microbiota in Gut–Brain Communication”, by Ygor Parladore Silva et al., had 1520 citations (34). This review outlines the current knowledge about the involvement of short-chain fatty acids (SCFAs) in microbiota–gut–brain interactions and highlights how future treatments for central nervous system (CNS) disorders can utilize SCFAs to regulate neuro-immunoendocrine functions, such as their effects on microglial maturation, neurotransmitter levels, and the pathogenesis of various CNS and metabolic disorders. The second most-cited paper, titled “Neuroglial Activation and Neuroinflammation in the Brain of Patients with Autism” (35), investigated whether immune-mediated mechanisms are involved in autism. An examination of the brain tissues and cerebrospinal fluid of autistic patients revealed significant neuroglial activation and neuroinflammation, especially in the cerebellum. Unique cytokine expression profiles were detected. These findings suggest that innate immune responses play a role in autism pathogenesis, which might guide future therapies. The third-ranked title, “Neuropathic Pain: A Maladaptive Response of the Nervous System to Damage,” by Michael Costigan, Joachim Scholz, and Clifford J. Woolf, examined the neurobiological mechanisms of neuropathic pain (36). A study revealed that neuropathic pain is a maladaptive response of the nervous system to damage involving multiple mechanisms, such as ectopic impulse generation, central sensitization, and neuroimmune interactions. Genetic factors also influence its development. Current treatments face challenges, and future research directions are proposed to better understand and manage these conditions.

#### 3.3.2 References cocitation analysis

**Figure 5:**
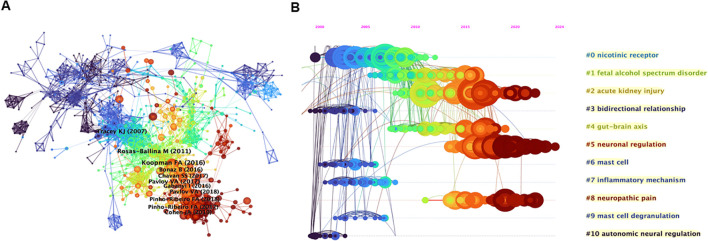
**(A)** References cocitation analysis generated by CiteSpace. **(B)** Clustering and timeline map of reference cocitation analysis.

To investigate the foundational themes of neuroimmune modulation, we performed a reference co-occurrence analysis via CiteSpace. The resulting network comprises 965 nodes and 4198 links, with the top 10 most co-cited articles identified (**Figure 5A**). Notably, three of these articles have been co-cited more than 80 times, underscoring their importance in the field (27, 44–52). The most co-cited article, by Frieda A. Koopman et al. (2016), with 122 co-citations, focused on rheumatoid arthritis (RA) (44). This study aimed to determine whether vagus nerve stimulation inhibits TNF-α production and affects RA disease severity. It included epilepsy patients and two cohorts of RA patients. Vagus nerve stimulation was applied, and the study revealed that it inhibited TNF-α production and significantly attenuated RA disease severity. The second most cocited article with 91 co-citations was published by Mauricio Rosas-Ballina et al. (2011) (45). This research aimed to determine how the neural circuit of the inflammatory reflex terminates cholinergic signaling. Researchers have measured acetylcholine levels, studied the effect of vagus nerve stimulation in nude mice, and used ChAT(BAC)-EGFP mice to identify acetylcholine-producing T cells. The results showed that vagus nerve stimulation increases acetylcholine release in the spleen, that T cells are required for the inflammatory reflex, and that acetylcholine-producing memory T cells are integral to the inflammatory reflex. The third article, by Kevin J. Tracey (2007), received 81 co-citations (46). This review explores the physiology and immunology of the cholinergic anti-inflammatory pathway (CAP). Research has focused on how the nervous system, specifically via the vagus nerve’s inflammatory reflex, can regulate cytokine release to prevent tissue injury. The CAP, which is activated by vagus nerve stimulation or cholinergic agonists, can inhibit cytokine synthesis and protect against cytokine-mediated diseases in various experimental models. As seen from the wide-ranging research in this area, the regulation of cytokines and the role of the nervous system in inflammation have been important topics for researchers, and many studies have explored the potential therapeutic applications of modulating the CAP. The three highly co-cited publications focused on the interaction between the nervous system and the immune system. They explored how the vagus nerve and related neural mechanisms regulate cytokine production in diseases. One study revealed that vagus nerve stimulation benefits rheumatoid arthritis patients. Another study revealed the role of acetylcholine-synthesizing T cells in the vagus nerve circuit. The latter details the CAP. These findings suggest potential new therapies for inflammatory and autoimmune diseases.

**Figure 5B** presents a timeline illustrating temporal changes in nodes, links, and clusters postclustering. The references were organized into eleven primary clusters, ranked by size: nicotinic receptor, fetal alcohol spectrum disorder (FASD), acute kidney injury (AKI), bidirectional relationship, gut–brain axis, neuronal regulation, mast cell, inflammatory mechanism, neuropathic pain, mast cell degranulation, and autonomic neural regulation. As shown in **Figure 4B**, the two largest clusters, nicotinic receptor and FASD, dominated research before 2013 and 2016, respectively. In contrast, contemporary research has focused on AKI, neuronal regulation, and neuropathic pain, which are emerging as current hotspots.

#### 3.3.3 Analysis of references with citation bursts

**Figure 6:**
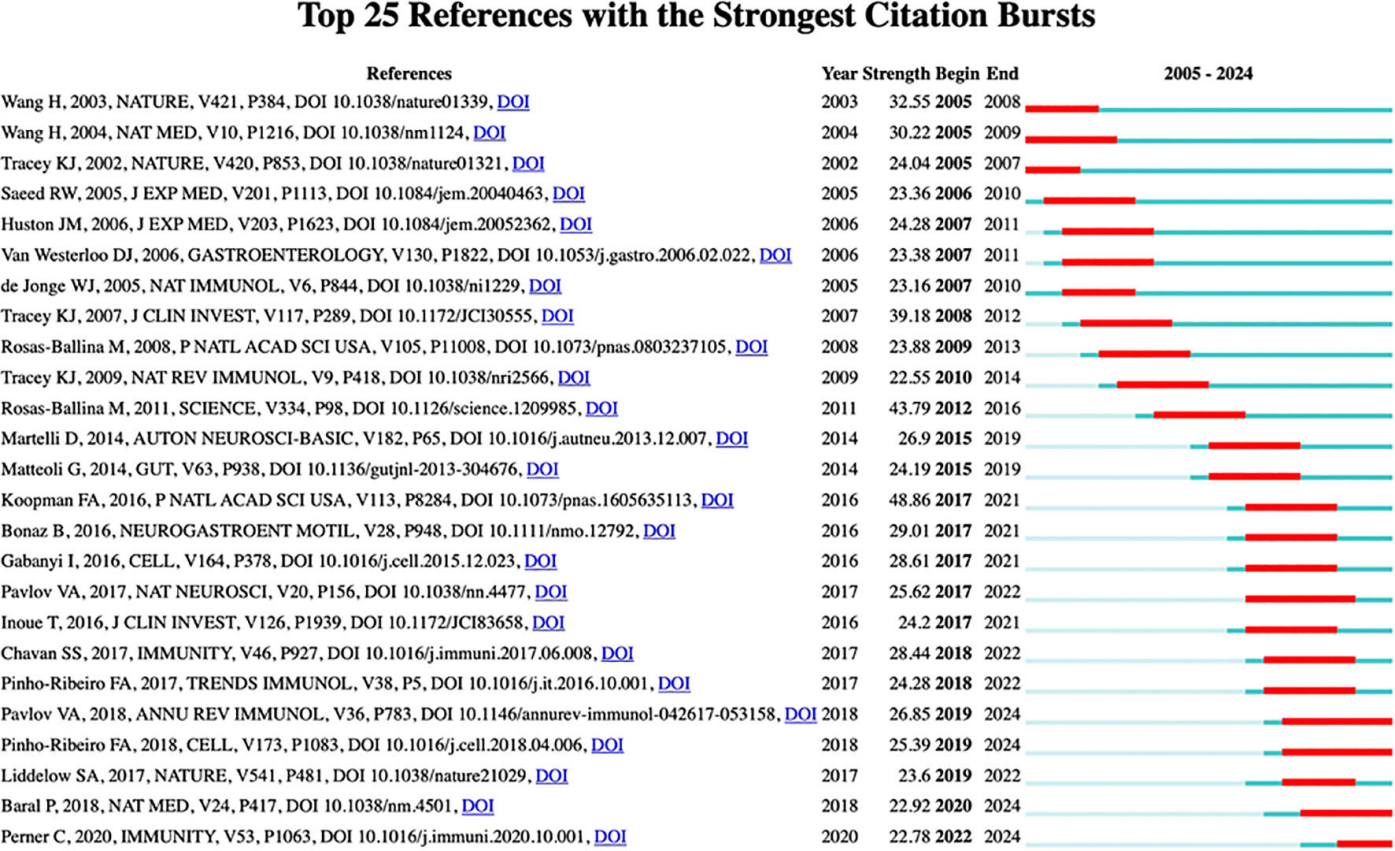
Keyword burst detection in the neuroimmune modulation field.

Burst detection, a method developed by Kleinberg, is widely recognized for identifying abrupt increases in the popularity of references or keywords within specific timeframes. This algorithm enables efficient tracking of concepts or topics that gain traction during defined periods. In our study, CiteSpace’s burst detection feature was employed to pinpoint pivotal references in neuroimmune modulation research. **Figure 6** shows the top 25 references with the most substantial citation bursts, where the blue lines denote temporal spans and the red segments highlight periods of intense citation activity. Notably, the two references with the highest burst values—48.86 and 43.79—were previously discussed in the context of co-citation frequency (44, 45). While the majority of reference bursts have diminished, four references exhibit persistent bursts, signaling sustained scholarly interest in these topics (48, 51, 53, 54). The four papers collectively investigated the complex interplay between the nervous and immune systems. For example, “Molecular and Functional Neuroscience in Immunity” systematically examined the mechanisms by which neural pathways modulate immune responses and inflammation (48). This paper elucidates a bidirectional communication pathway: sensory neurons detect immune-related molecules and pathogens, transmitting signals to the central nervous system (CNS), which subsequently modulates immune activity via efferent autonomic neurons. Notably, the involvement of the vagus nerve in the inflammatory reflex and the concept of an immunological homunculus were highlighted as groundbreaking insights, offering therapeutic potential for inflammatory disorders. In parallel, “Blocking Neuronal Signaling to Immune Cells Treats Streptococcal Invasive Infection” revealed that *Streptococcus pyogenes* exploits nociceptor neurons by releasing streptolysin S, triggering pain and inducing the release of calcitonin gene-related peptide (CGRP). This neuropeptide suppressed neutrophil recruitment and bactericidal function, identifying neural signaling as a critical target for combating invasive infection (51). Blocking this neuroimmune signaling with Botulinum neurotoxin A or a CGRP antagonist effectively treats infection, highlighting a new therapeutic approach. “Nociceptor sensory neurons suppress neutrophil and γδ T-cell responses in bacterial lung infections and lethal pneumonia” reveals that TRPV1+ nociceptors in the lung suppress protective immunity against *Staphylococcus aureus* pneumonia (53). Ablating these neurons improves survival, cytokine induction, and bacterial clearance by enhancing neutrophil and γδ T-cell responses. The neuropeptide CGRP, released by nociceptors, plays a crucial role in this immunosuppression. “Substance P Release by Sensory Neurons Triggers Dendritic Cell Migration and Initiates the Type-2 Immune Response to Allergens”, showing that allergens activate TRPV1+ sensory neurons, leading to Substance P release (54). This stimulates CD301b+ dendritic cells through MRGPRA1, promoting their migration to the draining lymph node and initiating the type-2 immune response. Sensory neurons thus act as primary allergen sensors crucial for allergic immune activation.

#### 3.3.4 Analysis of keywords

**Figure 7:**
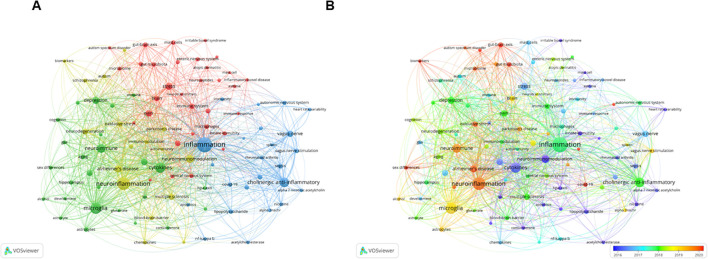
Visualized mapping of keyword co-occurrence analysis in clusters **(A)** or in years arranged (**B**, from 2004–2024) via VOSviewer.

Keywords are crucial indicators of the main topics and core content of a specific subject (55). In bibliometrics, keyword co-occurrence analysis is a common method for identifying research hotspots. This analysis assesses the relationships between keywords on the basis of their simultaneous appearance in documents (56). In our study, we extracted author keywords from 5,280 publications and analyzed them via VOSviewer. After the synonymous terms were consolidated, we identified 99 keywords that appeared at least 30 times. **Table 3** lists the 20 most frequent keywords. “Inflammation” is the most frequently occurring keyword, and the keywords with more than 200 co-occurrences include “neuroinflammation”, “microglia”, “cytokines”, “cholinergic anti-inflammatory pathway” and “neuroimmune”. The VOSviewer co-occurrence map (**Figure 7A**) groups all keywords into four clusters depicted in different colors. Furthermore, VOSviewer color-coded all keywords on the basis of their average year of appearance (**Figure 7B**). Earlier keywords are shown in blue, whereas more recent keywords appear in red.

**Table 3:** Top 20 keywords in terms of co-occurrences.

Rank	Keyword	Counts	Rank	Keyword	Counts
1	inflammation	710	11	vagus nerve	134
2	neuroinflammation	495	12	cytokine	131
3	microglia	402	13	pain	129
4	cytokines	325	14	multiple sclerosis	114
5	cholinergic anti-inflammatory pathway	292	15	neuropathic pain	112
6	neuroimmune	253	16	neurodegeneration	104
7	neuroimmunomodulation	188	17	neuroimmunology	100
8	depression	165	18	immune system	95
9	stress	162	19	oxidative stress	91
10	alzheimer's disease	153	20	brain	87

### 3.4 Clinical practices of neuroimmune modulation

**Figure 8:**
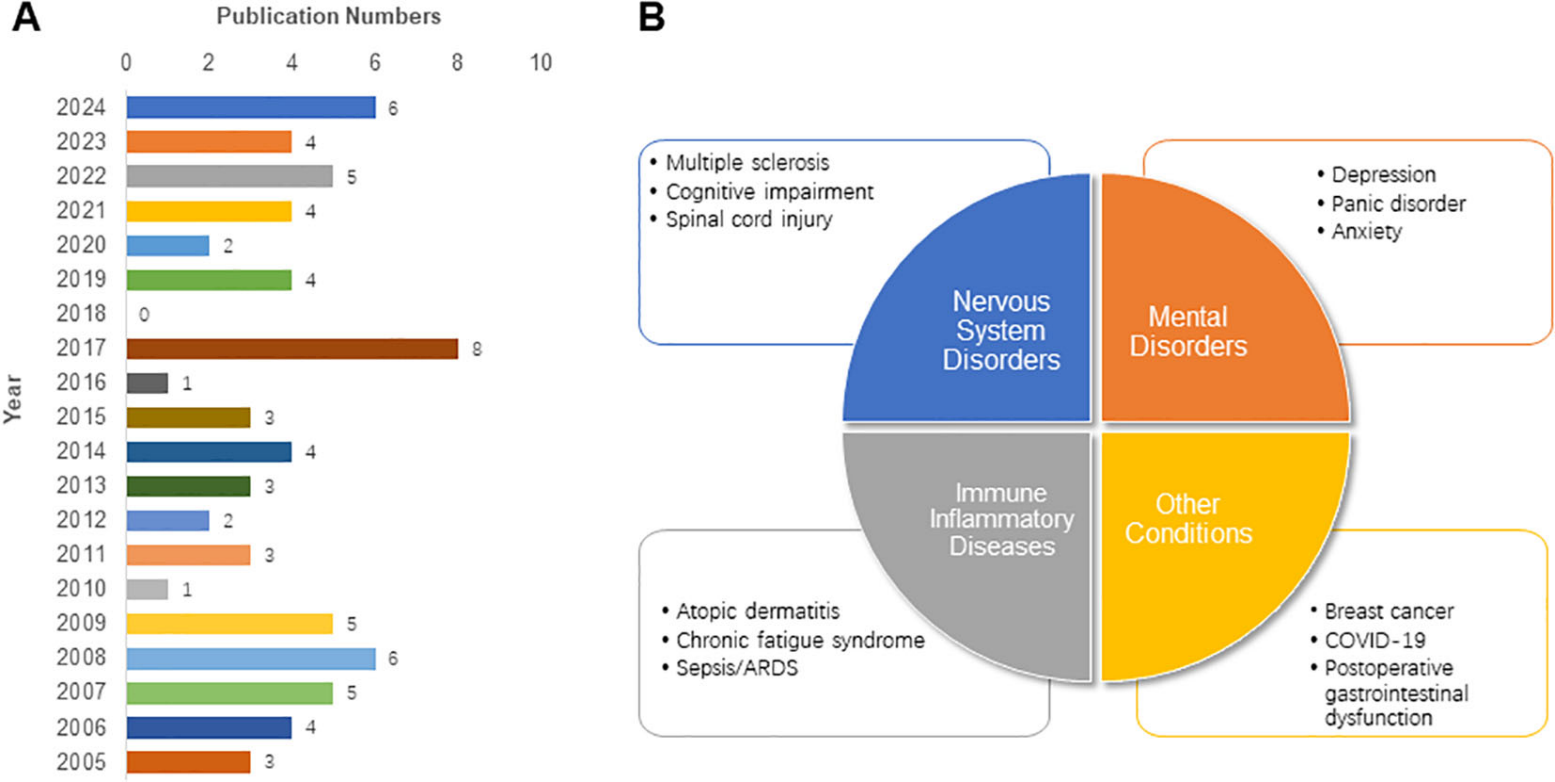
The annual number **(A)** and categories **(B)** of published clinical trials on neuroimmune modulation.

To further explore the current situation of targeting neuroimmune modulation for treating diseases clinically, we performed a literature search with the same topic and timeline in the PubMed database. A total of 73 published clinical trials were identified in the field of neuroimmune modulation (**Figure 8A**). On the basis of these trials, we categorized the diseases under investigation into four groups: nervous system disorders (such as multiple sclerosis (57, 58) and cognitive impairment (59, 60)), mental disorders (such as depression (61)), immune-inflammatory diseases (such as atopic dermatitis (62)), and other conditions (including breast cancer (63) and postoperative gastrointestinal dysfunction (64)) (**Figure 8B**). Among these trials, the inventions include neural modulation (such as transcutaneous vagus nerve stimulation (tVNS) to reduce cytokines (65)), drugs (such as vitamin D to evaluate IL-10 (57)) and comprehensive interventions (such as exercise to prevent migraine (66)) (**Table 4**). Most of the inventions were based on the mechanism of the CAP and the dynamic balance of cytokines, which was consistent with the above most frequent keywords. Although we did not apply a scale to comment on the trials we recruited, researchers should note that all these published trials had limitations such as relatively small sample sizes. Several trials had a short duration, which was finished by collecting the serum sample in one day.

**Table 4:** Inventions of clinical trials related to neuro-immune modulation.

Category	Inventions	Disease/target	Title	Journal	Year
Neural modulation	Transcutaneous vagus nerve stimulation	Healthy people, test cytokines and Chemokines	Noninvasive Transcutaneous Vagus Nerve Stimulation Decreases Whole Blood Culture-Derived Cytokines and Chemokines: A Randomized, Blinded, Healthy Control Pilot Trial	Neuromodulation	2016
	Breath training	Panic disorder	Effect of a slow-paced breathing with heart rate variability biofeedback intervention on pro-inflammatory cytokines in individuals with panic disorder - A randomized controlled trial	J Affect Disord	2023
		COVID-19	A randomized clinical trial to stimulate the cholinergic anti-inflammatory pathway in patients with moderate COVID-19-pneumonia using a slow-paced breathing technique	Front Immunol	2022
Drugs	Vitamin D	Multiple sclerosis	Short-term effect of high-dose vitamin D on the level of interleukin 10 in patients with multiple sclerosis: a randomized, double-blind, placebo-controlled clinical trial	Neuroimmunomodulation	2015
	Mitoxantrone	Multiple sclerosis	In vivo effects of mitoxantrone on the production of pro- and anti-inflammatory cytokines by peripheral blood mononuclear cells of secondary progressive multiple sclerosis patients	Neuroimmunomodulation	2006
	Nicotine	Postoperative ileus	Nicotine chewing gum for the prevention of postoperative ileus after colorectal surgery: a multicenter, double-blind, randomised, controlled pilot study	Int J Colorectal Dis	2017
Drugs	Physostigmine salicylate	Sepsis	Adjunctive use of physostigmine salicylate (Anticholium®) in perioperative sepsis and septic shock: study protocol for a randomized, double-blind, placebo-controlled, monocentric trial (Anticholium® per Se)	Trials	2017
	Dexmedetomidine	Postoperative delirium	In a secondary analysis from a randomised, double-blind placebo-controlled trial Dexmedetomidine blocks cholinergic dysregulation in delirium pathogenesis in patients with major surgery	Sci Rep	2023
	Theanine with sertraline	Depression	l-theanine adjunct to sertraline for major depressive disorder: A randomized, double-blind, placebo-controlled clinical trial	J Affect Disord	2023
	Vitamin B12 with antipsychotic Drugs	Alzheimer Disease	Vitamin B12 in Association with Antipsychotic Drugs Can Modulate the Expression of Pro-/Anti-Inflammatory Cytokines in Alzheimer Disease Patients	Neuroimmunomodulation	2017
Comprehensive therapy	Exercises	Migraine	Exercise-Induced Change in Plasma IL-12p70 Is Linked to Migraine Prevention and Anxiolytic Effects in Treatment-Naïve Women: A Randomized Controlled Trial	Neuroimmunomodulation	2017
	Psychological intervention	Cognitive impairment	The Managing Cancer and Living Meaningfully (CALM) Intervention Alleviates Chemotherapy-Related Cognitive Impairment in Patients with Breast Cancer by Modulating Pan-Immune-Inflammation Values	Integr Cancer Ther	2022
	Inhaled steroids	Asthma (children)	Adrenal function improves in asthmatic children on inhaled steroids: a longitudinal study	Neuroimmunomodulation	2006

## 4 Discussion

### 4.1 Primary findings

This study conducted visualized knowledge mapping on the basis of 5,280 publications and 73 clinical practices related to neuroimmune modulation over the past two decades. Since 2005, the number of publications related to neuroimmune modulation has steadily increased. The United States leads in both publication quantity and quality. Among institutions, Harvard University has the highest output. The most influential authors in this field include Maes, Michael, Kevin J. Tracey, and Terrence Deak. Acute kidney injury, neuronal regulation and neuropathic pain are current hotspots in the neuroimmune modulation field. “Inflammation”, together with “microglia”, “cytokines” and “cholinergic anti-inflammatory pathway”, were the most frequent author keywords. The findings from clinical practice highlight the potential of targeting neuroimmune modulation in the future.

### 4.2 Results of the study in context

#### 4.2.1 The current academic landscape of neuroimmune modulation research

In the academic landscape of neuroimmune research, the United States dominates both the quantity and quality of publications. Additionally, institutions in the United States, such as Harvard University, reach the highest rank. The U.S. leading in the current research field reflects its powerful technological strength due to its economic volume. Additionally, such research in the neuroimmune field has provided novel topics and explored new research directions for further research. Notably, China has emerged as a significant contributor, likely owing to its economic growth and strategic scientific policies. However, academic silos remain prevalent. Cite space-derived betweenness centrality (BC) scores reveal low connectivity across countries, institutions, and researchers, reflecting limited collaboration. Addressing these barriers to foster global cooperation remains a critical challenge for the research community.

#### 4.2.2 Acute kidney injury and neuropathic pain are key focuses of neuroimmune research

In AKI, the neuroimmune axis plays a crucial role in pathogenesis and progression, acting as a complex communication network between the nervous system and the immune system. Dysregulation of this crosstalk exacerbates kidney damage through inflammation, cell death, and impaired repair. On the one hand, overaction of the sympathetic nervous system leads to the release of catecholamines from sympathetic nerve endings and the adrenal medulla (67, 68). Then, catecholamines bind primarily to β-adrenergic receptors (β-ARs) on immune cells, which can stimulate the release of proinflammatory cytokines, increase neutrophil recruitment and activation, and modulate T-cell responses toward a more proinflammatory phenotype (67, 68). On the other hand, inflammatory signals can activate efferent vagus nerve fibers, and acetylcholine (ACh) is released from vagus nerve terminals in peripheral organs and potentially near renal nerves. The CAP is a major counterregulator of inflammation. Impairment of the CAP exacerbates AKI. Conversely, vagus nerve stimulation (VNS) or α7nAChR agonists are protective in experimental AKI models (69–71). In conclusion, stimulating the release of cytokines from both the sympathetic and parasympathetic nervous systems modulates immune cell function by releasing neurotransmitters that bind to specific receptors, with effects ranging from protective to deleterious. Balancing these autonomic pathways through pharmacological or nonpharmacological interventions or by targeting specific neurotransmitters or receptors requires further investigation to mitigate renal damage during AKI.

For neuropathic pain, neuroimmune crosstalk involves both the peripheral and central nervous systems. Nerve injury triggers neurotransmitter release, recruiting distinct immune cell populations, such as macrophages, neutrophils and mast cells. At the peripheral level, several cytokines, including TNF-α and IL-6, can directly activate nociceptive neurons (72–74). Moreover, infiltrating T cells can secrete IFN-γ to activate the STAT3 pathway in DRG neurons, leading to sustained hyperalgesia (75). At the central level, glial cells dominate central sensitization in the spinal cord and affect cortical and descending modulatory systems. In the spinal cord, microglia can receive signals such as CCL2 from the periphery, leading to the release of inflammatory mediators (76). Astrocytes can release IL-17 and S100β to persistently increase NMDA receptor activity and cause central sensitization (77, 78). Additionally, infiltrating T cells, such as CD4^+^ T cells, can secrete IL-17a to activate microglia/astrocytes and amplify inflammation, while T_reg_ cell dysfunction leads to the loss of pain suppression (79, 80). In the brain, microglia release IL-1β to inhibit NE neurons, thus decreasing the degree of inhibition (81). Notably, sex disparities in pain responses underscore the complexity of this neuroimmune interplay (82). Targeting neural or immune components holds promise for novel therapeutic strategies against these recalcitrant conditions.

#### 4.2.3 Four hotspots in neuroimmune studies and future directions

Notably, the cluster study by keyword co-occurrence map presented four colors, which indicated that the hotspots in neuroimmune studies could be categorized into four areas. We summarized these four areas as, first, the interaction between neuroimmune mechanisms and the gut microbiota and microbiome, as well as their effects on diseases such as asthma, atopic dermatitis, autism spectrum disorder, and inflammatory bowel disease. Second, the role and mechanism of the neuroimmune process in behaviors such as anxiety, depression and pain are influenced by factors such as aging and alcohol. Third, the associations between the acetylcholine–vagus nerve–immune–inflammatory axis and diseases as well as autonomic nerve function have been explored. Fourth, the role of neuroimmune-related mechanisms and their biomarkers in neurological diseases such as Alzheimer’s disease, autism, and Parkinson’s disease, as well as autoimmune diseases, should be explored.

The microbiota and microbiome have recently become popular topics in the academic field, and they are referred to as the second brain. However, how they affect the neuroimmune system in diseases remains largely unknown. Hong et al. revealed a beneficial shift in the gut microbiota composition in neuropathic pain treatment with reduced inflammatory cytokines (83). Li et al. reported that the gut microbiota may influence Alzheimer’s disease (AD) pathogenesis by modulating host homeostasis via a self-reinforcing mechanism involving immune signaling (84). Future works on how the microbiota forms a bidirectional regulatory network through the metabolite–immune–neural axis under different conditions might provide new insight for treating diseases.

With respect to behaviors such as anxiety, depression and pain, neuroimmune mechanisms are undoubtedly the research frontiers mentioned above. Recent studies have shown that peripheral immune-to-brain signaling can disrupt neural homeostasis via cytokines such as IL-6 and TNF-α and lead to depression (85). Moreover, inflammation-driven neuroendocrine-immune axis dysregulation, such as HPA axis hyperactivation and monoaminergic system suppression, can drive various abnormal behaviors (86–88). The development of BBB-penetrating anti-inflammatory drugs or the identification of personalized immune profiles for precision treatment might be considered in future studies.

In the context of the cholinergic axis, the CAP itself is a major counterregulator of inflammation. Targeting this axis, such as enhancing CAP, is expected to become a new direction for disease treatment, but organ-specific challenges in clinical translation need to be addressed. For neurological/autoimmune diseases, mechanisms and biomarkers are research frontiers. Peripheral-central immune crosstalk is undoubtedly the core mechanism of such diseases. For example, T cells can cross the BBB and attack myelin via MMP-9, together with B-cell-derived IL-6, leading to astrocyte reactivity and thus resulting in demyelination in multiple sclerosis (89). Several biomarkers, such as neurofilament light (NfL), can reflect the severity of axonal damage or be used to monitor treatment response in MS patients (90, 91). Multiomics integration, such as combining CSF proteomics and PET-MRI, could help to define a neuroimmune activity index for treating patients.

Taken together, these four areas encompass the majority of nervous system diseases, with neuroimmune mechanisms as the central theme. Unraveling the underlying molecular and cellular pathways governing these interactions could deepen our comprehension of neuroimmune dynamics, thereby facilitating the development of targeted therapeutic strategies for these conditions.

#### 4.2.4 Clinical trials on neuroimmune modulation remain a challenge

We also performed clinical trials on neuroimmune modulation and reported that most current studies focus on conditions such as multiple sclerosis, depression, and chronic fatigue syndrome. Treatment approaches encompass neural regulation, pharmacological interventions and comprehensive therapy. These investigations collectively highlight the core mechanisms of neural–immune interactions, particularly the CAP and cytokine balance. However, several challenges, including small sample sizes in published trials, outcome measurement challenges and gaps between animal and human disconnection, remain. Contrary results were reported between trials and basic studies, such as transcutaneous vagus nerve stimulation (tVNS), which led to an increase in inflammatory cytokines in both young and elderly individuals (92). Although the authors account for the above phenomenon resulting from psychological stress, the work left the question of whether it is more complicated in the clinical situation. Furthermore, Tina et al. reported that tVNS has no anti-inflammatory effect on diabetes, which is known to be associated with chronic inflammation (93). All these trials raise the challenge that targeting neuroimmune modulation in different diseases requires further verification in the future.

Future trials may explore several avenues, including expanding the disease spectrum beyond typical neuroimmune disorders to include conditions such as Alzheimer’s disease and irritable bowel syndrome; innovating treatment methods such as precise neural regulation technologies, with noninvasive approaches such as transcranial magnetic stimulation warranting investigation; leveraging biomarkers for treatment efficacy assessment; addressing clinical translation challenges, including individual variability in treatment response and the need for personalized strategies incorporating genetic typing; overcoming limitations of predominantly short-term trials through longitudinal safety and efficacy data; and advancing interdisciplinary integration, as evidenced by combined psychological–neural interventions in cancer patients who simultaneously improve psychological and immune parameters, indicating a promising research direction.

### 4.3 Limitations of the current bibliometric study

The current study has several limitations. First, although the current study recruited only references from the WOSCC database and PubMed, the main bibliometric analysis was conducted on the basis of WOSCC data style. The main reason was that CiteSpace was built on the basis of WOS style. Second, references in the English language were included, which might undervalue the contributions from non-English publications. Third, we did not perform a sensitivity or specificity check for the search results. Fourth, the quality of the references was ranked by the software on the basis of the indexes and the time at which we conducted the analysis. For example, the number of citations of a certain reference could be updated rapidly, which might influence burst detection. Fifth, we recruited only published clinical trials from PubMed, which raises the risk of missing key studies from other clinical trial registry sources, such as ClinicalTrials.gov or the European Clinical Trials Registry. Sixth, we did not apply a scale to evaluate the quality of the clinical research, which might cause bias. Because our original purpose was to provide a visualized knowledge map of neuroimmune studies, future works evaluating neuroimmune trials could be carried out in the future.

## 5 Conclusions

This analysis offered knowledge mapping in the current research field of neuroimmune modulation. Mechanistic studies combined with neuronal regulation are currently popular in the field. Targeting neuroimmune crosstalk might provide new opportunities to treat nervous system diseases. The current analysis has provided valuable information on future directions within the field.

## Data Availability

The original contributions presented in the study are included in the article/supplementary material. Further inquiries can be directed to the corresponding author/s.
